# Functional cerebello–cortico–limbic connectivity in aggression: A resting-state 7T fMRI study in healthy volunteers

**DOI:** 10.1162/IMAG.a.42

**Published:** 2025-06-20

**Authors:** Elze M.L. Wolfs, Jana Klaus, Dennis J.L.G. Schutter

**Affiliations:** Department of Experimental Psychology, Helmholtz Institute, Utrecht University, Utrecht, The Netherlands

**Keywords:** aggression, cerebellum, functional connectivity, impulsivity, intrinsic networks, resting-state functional magnetic resonance imaging, steroid hormones

## Abstract

Structural and functional magnetic resonance imaging (fMRI) studies of the human brain point towards the involvement of the cerebellum in aggressive behaviour. However, the extent to which the cerebellum is part of the brain’s intrinsic network subserving aggression remains unknown. To address this issue, 28 healthy volunteers aged 18–32 years underwent a 9-min resting-state 7T fMRI scan, and functional connectivity between the posterior vermis, fastigial nuclei (FN), bilateral Crus I–II of the cerebellum and the amygdala, hypothalamus, subgenual anterior cingulate cortex (sgACC), and ventromedial prefrontal cortex was examined. In addition, behavioural and self-reported indices of aggression and basal steroid hormone levels were assessed and correlated to resting-state cerebellar functional connectivity. Results demonstrated that the posterior vermis was functionally connected to the hypothalamus, centromedial amygdala (CMA), and sgACC. The FN showed functional connections with the CMA and hypothalamus. Bilateral resting-state activity of Crus I–II was significantly associated with resting-state activity of the left sgACC. Functional connectivity of the posterior vermis and FN with the hypothalamus, CMA, and left sgACC was significantly correlated with impulsivity and aggressive behaviour. Associations between cortisol and FN–hypothalamus functional connectivity and between testosterone and cerebellum–sgACC functional connectivity were observed. The findings show that the cerebellum and its connections are part of an intrinsic subcortical motivational circuit associated with aggression.

## 1 Introduction

Evidence from neuropsychological, structural, and task-based functional MRI studies suggests that the deep cerebellar nuclei, posterior vermis, and posterolateral lobules of the cerebellum are implicated in non-motor-related affective and cognitive aspects associated with anger and aggression (Bertsch et al., 2022;Klaus & Schutter, 2021;Wolfs, van der Zwaag, et al., 2023). In fact, the cerebellum is proposed to be an integral part of the neural network of anger and aggressive behaviour composed of core regions that include the amygdala, hypothalamus, periaqueductal grey (PAG), and prefrontal cortex (for a review seeKruithof et al., 2022). The basolateral (BLA) and centromedial (CMA) amygdala are implicated in threat appraisal and responsivity of the autonomic and somatomotor systems, respectively (Bzdok et al., 2013;Göttlich et al., 2022;Terburg et al., 2018). The hypothalamus receives input from the amygdala and is involved in preparing the body for action through activation of the peripheral nervous system and signalling the release of hormones, such as testosterone and cortisol (Dampney, 2011;LeDoux, 2012;Lischinsky & Lin, 2020). The steroid hormones cortisol and testosterone are the end products of the hypothalamic-pituitary-adrenal- and hypothalamic-pituitary-gonadal-axis, respectively (Johnson et al., 1992). Both hormones play a role in aggression and are involved in approach behaviour and fearful withdrawal (Montoya et al., 2012). In particular, the ratio between testosterone and cortisol has been proposed to be a marker for aggressive behaviour (Popma et al., 2007;Terburg et al., 2009;Van Honk et al., 2010). The hypothalamus and PAG are suggested to contribute to the onset of autonomic and emotional changes and mediate subcortically instigated aggression (Panksepp & Biven, 2012). Electric stimulation of the PAG in animals, for instance, can elicit rage-like behaviour, while the orbital frontal (OFC) and ventromedial prefrontal cortex (vmPFC) serve a regulatory role in controlling impulsivity, anger, and aggressive behaviour (Mehta & Beer, 2010;Panksepp & Zellner, 2004).

The study of large-scale organized brain activity in the absence of task-related engagement can provide insights into endogenous connectivity dynamics of the brain that ultimately govern task-related activity and behaviour (Fröhlich, 2016). Thus far, for the cerebellum, intrinsic resting-state networks akin to those found in the cerebral cortex have been described (e.g.,Buckner et al., 2011;Habas, 2021;Habas et al., 2009;Krienen & Buckner, 2009;Marek et al., 2018). These include the default mode network, which is predominantly active during relaxed wakefulness and associated with self-referential processes (Raichle, 2015), and the salience network, which is linked to emotional regulation and reward processing (Gan et al., 2016;Seeley et al., 2007). Furthermore, resting-state activity of the posterior cerebellum is correlated with resting-state activity of the frontoparietal central executive network (Habas et al., 2009;O’Reilly et al., 2009). Several lines of evidence point towards deviant functional connectivity between the cerebellum and in particular the frontal areas of the central executive network in aggression. For example, reduced functional cerebello–frontal connectivity has been observed in Tourette disorder patients with rage attacks (Amaoui et al., 2022;Atkinson-Clement et al., 2020). Lower functional connectivity between the OFC, a region implicated in impulsivity and emotion regulation, and the cerebellum has also been reported in criminal and violent offenders (Leutgeb et al., 2016). A more recent study showed decreased functional connectivity between the deep cerebellar nuclei and the medial OFC in veterans with as compared to veterans without reactive aggression symptoms (Wolfs, van Lutterveld, et al., 2023). In contrast to reduced resting-state functional connectivity between the cerebellum and cerebral cortex, there is tentative evidence that functional connectivity between the cerebellum and subcortical areas is actually enhanced. Higher functional connectivity between the cerebellum and amygdala has been observed in violent offenders compared with non-violent controls (Leutgeb et al., 2016). In concordance, permutation-based cluster analyses showed a general pattern of increased functional connectivity between the motor-dedicated anterior cerebellum and the amygdala and anterior cingulate (limbic) cortex in combat veterans with impulsive aggression (Varkevisser et al., 2017). In another resting-state fMRI study, functional connectivity between posterior cerebellar lobule VI and the BLA was found to positively correlate with behavioural inhibition (Roy et al., 2014).

The discussed findings suggest that the cerebellum is part of the neural circuit of aggression. In the current study, we sought to extend previous results from our structural MRI and task-based fMRI studies (Wolfs, Klaus, et al., 2023;Wolfs, van der Zwaag, et al., 2023) to include the cerebellum in the intrinsic aggression network of the brain. The aim was to examine resting-state functional connectivity between the cerebellum and subcortical and cortical nodes of the brain’s aggression network using 7T resting-state fMRI in healthy volunteers. The regions of interest (ROIs) were selected based on previous structural and functional MRI studies that found associations of cerebellar areas with impulsivity and aggressive behaviour. These ROIs were the posterior vermis (Wolfs, Klaus, et al., 2023), fastigial nuclei (Wolfs, van Lutterveld, et al., 2023), as well as left Crus I and right Crus II of the posterolateral cerebellar hemispheres (Wolfs, van der Zwaag, et al., 2023). For the cortico-limbic ROIs, the hypothalamus, BLA, CMA, sgACC and vmPFC were selected. In addition, associations between functional cerebellar connectivity and self-reported impulsivity and physical aggression, task-based aggressive behaviour, and basal steroid hormone levels were explored.

## 2 Methods

### 2.1 Participants

Thirty healthy volunteers who participated in the same study as published inWolfs, van der Zwaag, et al. (2023)were included in the present study. Inclusion criteria were age between 18 and 35 years old, right handed, no current or history of psychiatric or neurological conditions, MRI compatibility (i.e., no claustrophobia, electronic implants, pregnancy, or metal in their body), and no use of psychotropic medication or recreational drugs. Volunteers received travel reimbursement and monetary compensation for participation. Written informed consent was obtained from each participant. The study was approved by the medical ethical committee of the University Medical Center Utrecht (NL77559.041.21) and performed in accordance with the Declaration of Helsinki. Of the 30 participants, one participant was excluded due to technical issues with the resting-state scan and one participant for excessive motion throughout the entire scanning session (>1 mm). This resulted in a final sample size of 28 participants.

### 2.2 Measures of aggressive behaviour

#### 2.2.1 Aggressive and impulsive traits

The Buss–Perry Aggression (BPA) questionnaire (Buss & Perry, 1992) was used to assess self-reported trait anger and aggression. Twenty-nine questions were answered on a 5-point scale ranging from “extremely uncharacteristic of me” to “extremely characteristic of me”. The Barratt Impulsiveness Scale (BIS-11) was administered to obtain a self-reported measure of trait impulsivity (Patton et al., 1995). Thirty questions were answered on a 4-point scale ranging from “seldom/never” to “almost always”. Both questionnaires were administered after the fMRI scanning session. Based on our previously observed correlations with cerebellar grey matter volume (Wolfs, Klaus, et al., 2023), the BPA subscale physical aggression and total BIS-11 score were selected for the present study.

#### 2.2.2 Steroid hormones

Saliva samples were collected before the MRI scanning session. Participants were instructed to not eat or drink anything besides water for two hours before the study. Immediately after sample collection through spitting in a saliva vial, the samples were stored in a freezer at -80°C. The Central Diagnostic Laboratory (CDL) at the University Medical Centre Utrecht determined testosterone levels via radio-immunoassay and cortisol levels via liquid chromatography-tandem mass spectroscopy. More details on the extraction of testosterone and cortisol levels from saliva can be found inWolfs, van der Zwaag, et al. (2023). Two participants with undetectable levels of cortisol (<0.5 nmol/L) were removed from all analyses including individual hormone levels.

#### 2.2.3 Task-based aggression

Volunteers who were included in the current study had also performed the Point Subtraction Aggression Paradigm (PSAP) after the resting-state fMRI scan (seeWolfs, van der Zwaag, et al., 2023). The PSAP is a validated computerized task which assesses aggressive behaviour towards a fictional opponent who steals points from the participant during a money earning game (Cherek et al., 1997). Aggressive behaviour was operationalized as the number of times that the participant steals from the opponent, either as a direct reaction to a steal by the opponent (reactive aggression) or not (proactive aggression) (Wolfs, van der Zwaag, et al., 2023). Importantly, points taken away from the opponent were not added to the number of points of the other player. Six participants showed no reactive aggression and four participants showed no proactive aggression, and were not included in the respective analyses.

### 2.3 Image acquisition and preprocessing

All images were acquired on a 7T Philips Achieva scanner (Philips, Best, The Netherlands) equipped with an 8Tx/32Rx rf coil (Nova Medical, Wilmington, USA). T1-weighted images were acquired with Magnetization Prepared 2 Rapid Acquisition Gradient Echoes (MP2RAGE,Marques et al., 2010; TE/TR/TR_mp2rage_= 2.5 ms/6.2 ms/5.5 s, SENSE_y/z_= 1.8/1.8, flip angle = 8/5º, TI = 800/2700 ms, voxel size = 0.8 × 0.8 × 0.8 mm, FOV = 230 × 230 × 186 mm, scanning time = 638 s). Resting-state functional images were acquired with 3D echo planar imaging (3D-EPI, TE/TR_vol_= 17 ms/1.3 s, SENSE_y/z_= 2.60/3.27, flip angle = 20^o^, voxel size = 1.8 × 1.8 × 1.8 mm, FOV = 200 × 200 × 176 mm, scanning time = 540 s). At intermediate resolutions, fMRI benefits strongly from the increased SNR and BOLD responses at 7T. BOLD responses increase approximately linearly with field strength (van der Zwaag et al., 2009) and this can facilitate much shorter acquisitions at 7T. Up to fourfold shorter imaging sessions can suffice when scanning at 7T (Cai et al., 2021). In addition, the acquisition parameters were matched with the two task-based fMRI runs performed in the same scanning session (Wolfs, van der Zwaag, et al., 2023). Scan time is inherently limited because of the natural attention span and motivation of the participants. Additionally, cerebellar responses tend to be smaller than cerebral BOLD responses (Boillat & van der Zwaag, 2019). Hence, we expected to benefit strongly from 7T imaging to reliably visualize these responses. To optimize 7T cerebellar signal, universal Kt-points pulses were used to reduce B1 inhomogeneity (Gras et al., 2017;Oliveira et al., 2021;Roos et al., 2019). After each 3D-EPI fMRI acquisition, five more volumes were acquired with reversed phase encoding direction to allow distortion correction.

In agreement with the study ofWolfs, van der Zwaag, et al. (2023), pre-processing of the NIfTI images was performed using FSL (FMRIB’s Software Library, Oxford, UK) v6.0.2 (Jenkinson et al., 2012). PAR/REC image files were converted to NIfTI with dcm2niix (Li et al., 2016). Structural T1-weighted data were obtained from the two inversion time images using MATLAB (The Mathworks, Inc;https://github.com/JosePMarques/MP2RAGE-related-scripts). Distortion correction was performed using FSL’s top-up (Andersson et al., 2003;Smith et al., 2004). Using fMRI Expert Analysis Tool v6.00 (FEAT;Woolrich et al., 2001), functional scans were motion corrected with MCFLIRT (Jenkinson et al., 2002), smoothed at 3 mm Full Width at Half Maximum (FWHM) with SUSAN (Smith & Brady, 1997), and a brain mask was created from the mean functional volume with the Brain Extraction Tool (BET;Smith, 2002). Functional data were normalized for higher-level analyses by a single scaling factor (“grand mean scaling”) and realigned to the structural scan and MNI152 standard space by affine registration using FLIRT (Greve & Fischl, 2009;Jenkinson & Smith, 2001;Jenkinson et al., 2002). ICA-AROMA (Pruim et al., 2015) was used to generate 100 independent components, which were extracted from the functional data based on spatial patterns, frequency spectra, and time series. The classification (signal vs. noise) of each component was checked manually and was reclassified if necessary (Griffanti et al., 2017), followed by ICA-AROMA’s non-aggressive denoising. Furthermore, cerebrospinal fluid (CSF) and white matter (WM) signals were extracted from the structural scans after FAST segmentation (Zhang et al., 2001). CSF and WM signals from the functional scans were used as nuisance regressors. Functional data were high-pass filtered at 0.01 Hz.

### 2.4 Regions of interest

Left Crus I, right Crus II, the posterior vermis, and the fastigial nuclei were selected as cerebellar ROIs. A 5 mm sphere was placed around MNI coordinates*x*= -17,*y*= -71,*z*= -29 for left Crus I/lobule VI, and around*x*= 42,*y*= -61,*z*= -49 for left Crus II. These task-based peak coordinates were previously found during provocations and during stealing in the PSAP, respectively (Wolfs, van der Zwaag, et al., 2023). Furthermore, the posterior vermis and fastigial nuclei have shown strong links to the limbic system and aggressive behaviour (e.g.,Jackman et al., 2020;Schmahmann et al., 2022;Wolfs, Klaus, et al., 2023;Wolfs, van Lutterveld, et al., 2023;Zanchetti & Zoccolini, 1954). For each participant, the posterior vermis and fastigial nuclei were individually and automatically segmented using the SUIT atlas (Diedrichsen, 2006;Diedrichsen et al., 2009).

Based on prior research on the neurobiological network of aggression, nine ROIs were selected from the amygdala, hypothalamus, and prefrontal cortex (Lischinsky & Lin, 2020;Rosell & Siever, 2015). Freesurfer (http://surfer.nmr.mgh.harvard.edu/) individual segmentations of the hypothalamus (Billot et al., 2020) and bilateral amygdala, including the BLA and CMA (Saygin et al., 2017), were used for each participant. For the hypothalamic ROI, we exclusively focused on the superior tubular region due to signal drop out of the other hypothalamic nuclei. This region includes the dorsomedial nucleus and parts of the lateral hypothalamus and paraventricular nucleus which are implicated in sympathetic fight-or-flight responses (Todd & Machado, 2019) and violent behaviour (Haller, 2018). For the ROIs of the amygdala, the BLA comprised four subregions, that is the basal, lateral, paralaminar, and accessory basal nuclei. The CMA was defined as the central and medial nuclei of the amygdala. The PAG was not included as an ROI due to its small size and issues in proper localization of this midbrain structure. For the prefrontal cortex, the medial orbitofrontal cortex (mOFC) and vmPFC have been associated with impulse regulation and reward-related decision making (Mehta & Beer, 2010;Rolls et al., 2020). The rostral/subgenual anterior cingulate cortex (sgACC) is involved in processing signals related to reward and emotion (Etkin et al., 2011). In MNI space, bilateral prefrontal areas a24 and 10r were parcellated by the Glasser HCP multimodal atlas (Glasser et al., 2016) to represent functional regions of the sgACC and vmPFC, respectively. The mOFC could not be analysed due to signal drop out. ROIs were resampled to the functional scans (1.8 mm isotropic voxels).

### 2.5 ROI-to-ROI static functional connectivity

On the subject level, ROI-to-ROI static functional connectivity analysis was performed in CONN v22 (Nieto-Castanon, 2020;Nieto-Castanon & Whitfield-Gabrieli, 2022). For every participant, ROI-to-ROI connectivity matrices were estimated characterizing the functional connectivity between each pair of regions among 13 ROIs. Functional connectivity strength was represented by Fisher-transformed bivariate correlation coefficients from a general linear model (weighted-General Linear Model (GLM),Nieto-Castanon, 2020) and estimated separately for each pair of ROIs, characterizing the association between their BOLD signal time series. To compensate for possible transient magnetization effects at the beginning of each run, individual scans were weighted by a step function convolved with an SPM canonical haemodynamic response function and rectified.

Group-level analyses were performed using a GLM (Nieto-Castanon, 2020). For each individual connection, a separate GLM was estimated, with first-level connectivity measures at this connection as dependent variables. Connection-level hypotheses were evaluated using multivariate parametric statistics with random effects across subjects and sample covariance estimation across multiple measurements. Inferences were performed at the level of individual ROIs and based on parametric multivariate statistics, combining the connection-level statistics across all connections from each individual ROI (connection threshold*p*< 0.01). Results were thresholded using a false discovery rate corrected*p*_FDR_< 0.05 ROI-level threshold (Benjamini & Hochberg, 1995). While this analysis provides connectivity measures between all ROIs as output, we focused on the connectivity between the a priori selected cerebellar and (sub)cortical ROIs.

### 2.6 Brain–behaviour associations

Analyses were performed with R v4.3.1 in RStudio v2023.09.1 +494 for Windows (RStudio Team, 2018). Separate linear models were fitted to evaluate associations between static functional connectivity and behavioural indices of aggression. By-participant values for all significant functional connections served as independent variables. The behavioural indices served as dependent variables and consisted of the physical aggression score of the BPA and the total score of the BIS-11 (Wolfs, Klaus, et al., 2023), salivary steroid hormone levels testosterone (T) and cortisol (C), the T/C ratio, and task-based reactive and proactive aggressive behaviour (Wolfs, van der Zwaag, et al., 2023). Individual testosterone and cortisol levels were*Z*-transformed separately for males and females to account for sex-related differences in steroid hormone levels. The T/C ratio was calculated by dividing*Z*-transformed testosterone levels by*Z*-transformed cortisol levels. Proactive aggression was calculated as the number of key presses on the steal option corrected for the total number of key presses and the number of provocations, scaled by 1000 (i.e., [1000 × steal key presses]/[total key presses × provocations];Skibsted et al., 2017). Proactive aggression without provocation was determined as the percentage of steals (i.e., [steal key presses/total key presses] × 100).

The initial linear models included main effects for all observed significant static functional connectivity values. Stepwise regression using the*stepAIC*function in the*MASS*package (Venables & Ripley, 2002) was performed to identify the models most predictive of the respective behavioural measure. In the winning models, determined by the lowest AIC value, normality of residuals, absence of multicollinearity, and the presence of influential observations were examined using the*check_heteroskedasticity*,*check_multicollinearity*, and*check_outliers*functions in the*performance*package (Lüdecke et al., 2021). Statistical significance was set at <0.05 (two-tailed) with FDR correction applied for multiple comparisons within each model (Benjamini & Hochberg, 1995). No statistical correction was applied for the total number of models.

To check for the possible confound of motion, static functional connectivity values and measures of aggressive behaviour were correlated to the median framewise displacement (FD). FD values were calculated by averaging the rotation/translation parameter differences with matrix RMS formulation (Jenkinson et al., 2002). Average FD values of each participant were correlated with their static functional connectivity outcomes and measures of aggressive behaviour. No significant associations were observed (all*p*s ≥ 0.373).

## 3 Results

Descriptives of the study sample and averaged measures of aggressive behaviour are reported inTable 1.

**Table 1.** Descriptives and measures of aggressive behaviour in the study sample.

	*M* ( *SD* )	Range
Age (years)	22.96 (3.18)	18 – 32
Male ( *n* )	14 (50%)	
BPA physical aggression	17.11 (5.24)	10 – 29
BIS-11 total impulsivity	61.39 (8.96)	44 – 78
Testosterone (pmol/L)		
Males ( *n* = 12)	244.25 (44.69)	201.00 – 338.00
Females ( *n* = 14)	105.36 (49.16)	46.00 – 198.00
Cortisol (nmol/L)		
Males ( *n* = 12)	2.60 (1.34)	1.20 – 5.20
Females ( *n* = 14)	5.09 (5.26)	0.80 – 21.40
T/C ratio	-4.07 (22.87)	-115.69 – 4.67
PSAP reactive aggression ( *n* = 22)	2.00 (1.83)	0.31 – 8.71
PSAP proactive aggression ( *n* = 24)	13.36 (7.82)	0.89 – 35.38

Note: BIS-11 = Barratt Impulsiveness Scale; BPA = Buss–Perry Aggression questionnaire; PSAP = Point Subtraction Aggression Paradigm.

### 3.1 Resting-state functional connectivity

Significant functional connections between cerebellar and (sub)cortical ROIs are shown inFigure 1. Results showed functional connectivity between the vermis and hypothalamus (*T*_27_= 8.81,*p*< 0.001), left CMA (*T*_27_= 5.37,*p*< 0.001), and right CMA (*T*_27_= 3.91,*p*= 0.002). Additionally, the vermis showed anti-correlations to the prefrontal left sgACC (*T*_27_= -3.53,*p*< 0.004). The fastigial nuclei showed correlations to the hypothalamus (*T*_27_= 6.11,*p*< 0.001), left CMA (*T*_27_= 4.38,*p*< 0.001), and right CMA (*T*_27_= 5.76,*p*< 0.001). Anti-correlations to left sgACC were found for functional regions in left Crus I (*T*_27_= -3.50,*p*= 0.004) and right Crus II (*T*_27_= -2.93,*p*= 0.012). An overview of all results including the correlations between subcortical and prefrontal structures can be found in theSupplementary Materials (Table S1).

**Fig. 1.**
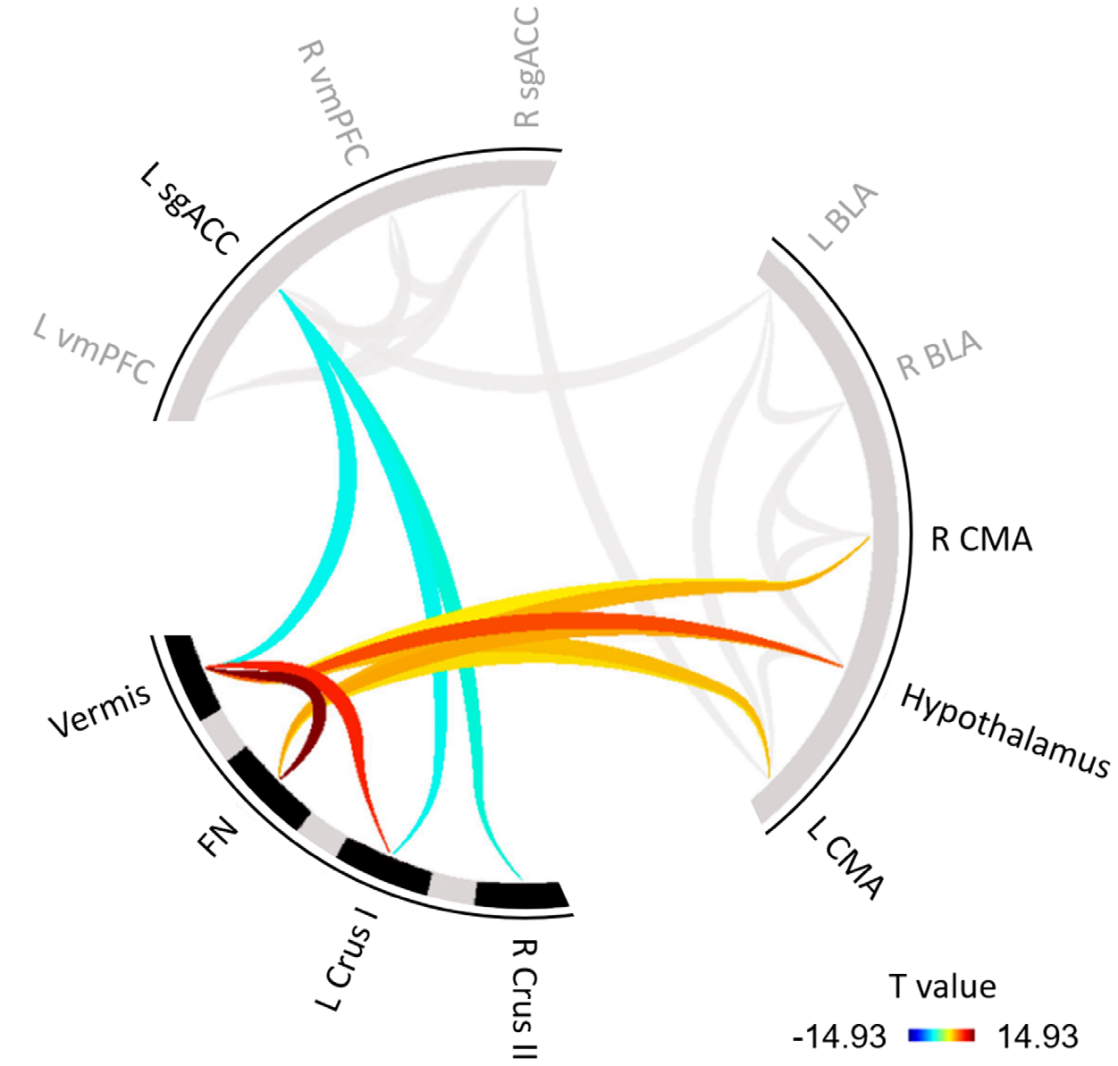
Static ROI-to-ROI functional connectivity between the cerebellum and cortico-limbic regions. Warm colours indicate positive associations and cold colours indicate negative associations. Darker values correspond to higher (absolute) correlations. BLA, basolateral amygdala; CMA, centromedial amygdala; FN, fastigial nuclei; L, left; R, right; sgACC, subgenual anterior cingulate cortex; vmPFC, ventromedial prefrontal cortex.

### 3.2 Brain–behaviour associations

The complete documentation of all models is given in the Supplementary Materials (Figs. S1-S5,Tables S2-S10).

#### 3.2.1 Aggressive and impulsive traits

**Fig. 2.**
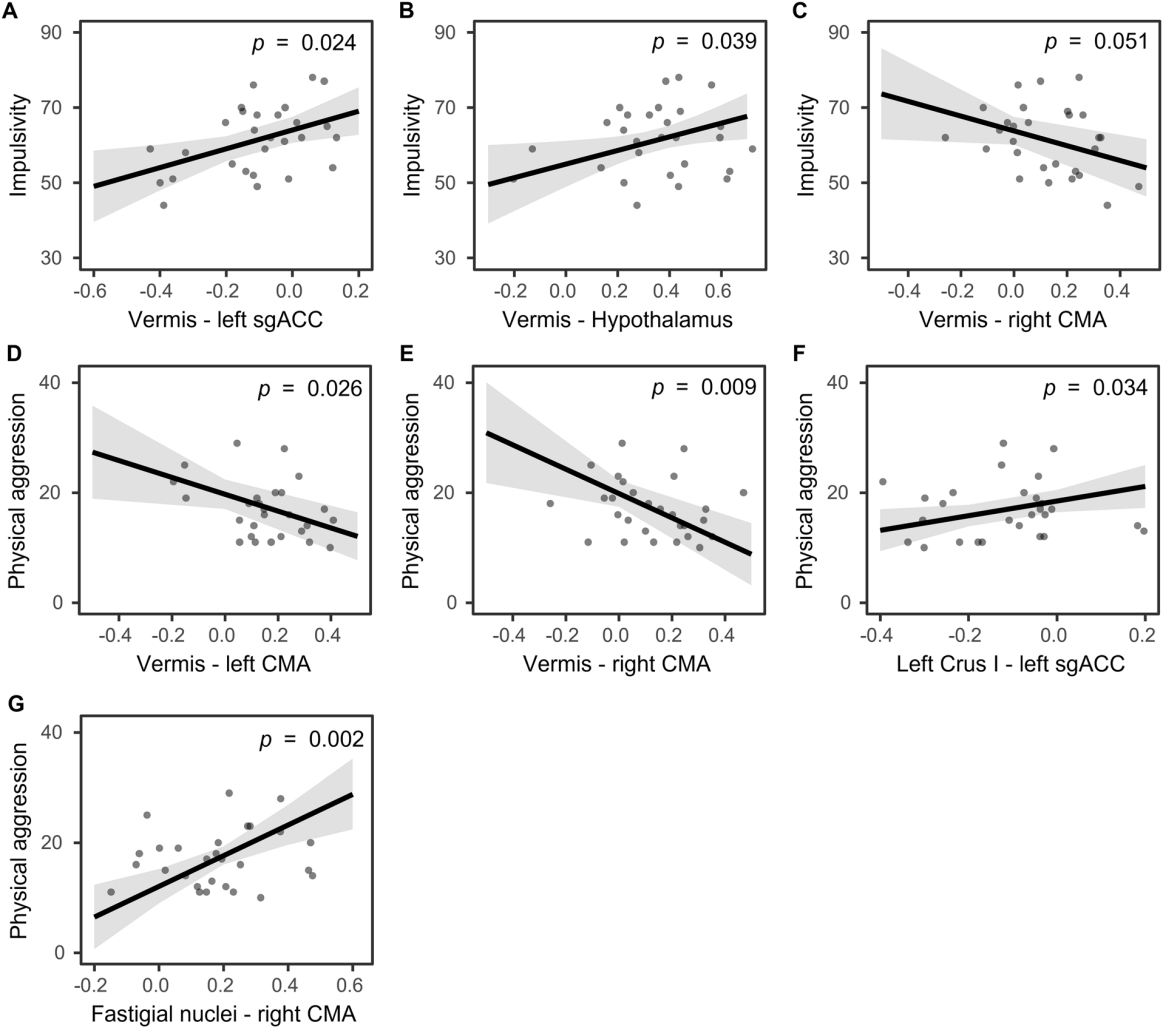
Significant associations between resting-state cerebello–limbic functional connectivity and impulsive (A-C) and aggressive (D-G) traits. Shaded areas illustrate standard errors. Each point indicates one participant.

Significant associations between aggressive and impulsive traits and functional connectivity are depicted inFigure 2. Higher self-reported impulsivity was associated with reduced negative functional connectivity between the vermis and the left sgACC (*β*= 25.01,*SE*= 8.87,*t*= 2.82,*p*= 0.024;Fig. 2A) and increased positive functional connectivity with the hypothalamus (*β*= 18.11,*SE*= 7.46,*t*= 2.43,*p*= 0.039;Fig. 2B). Moreover, there was a marginally significant negative association with functional connectivity between the vermis and the right CMA (*β*= -19.72,*SE*= 9.10,*t*= -2.17,*p*= 0.051;Fig. 2C). Higher self-reported physical aggression was correlated with lower functional connectivity between the vermis and left CMA (*β*= -15.30,*SE*= 5.95,*t*= -2.57,*p*= 0.026;Fig. 2D) and right CMA (*β*= -22.11,*SE*= 6.96,*t*= -3.18,*p*= 0.009;Fig. 2E). Conversely, higher self-reported physical aggression was associated with reduced negative functional connectivity between left Crus I and left sgACC (*β*= 13.25,*SE*= 5.64,*t*= 2.35,*p*= 0.034;Fig. 2F) and increased functional connectivity between the fastigial nuclei and right CMA (*β*= 27.91,*SE*= 5.64,*t*= 2.35,*p*= 0.002;Fig. 2G).

#### 3.2.2 Steroid hormones

**Fig. 3.**
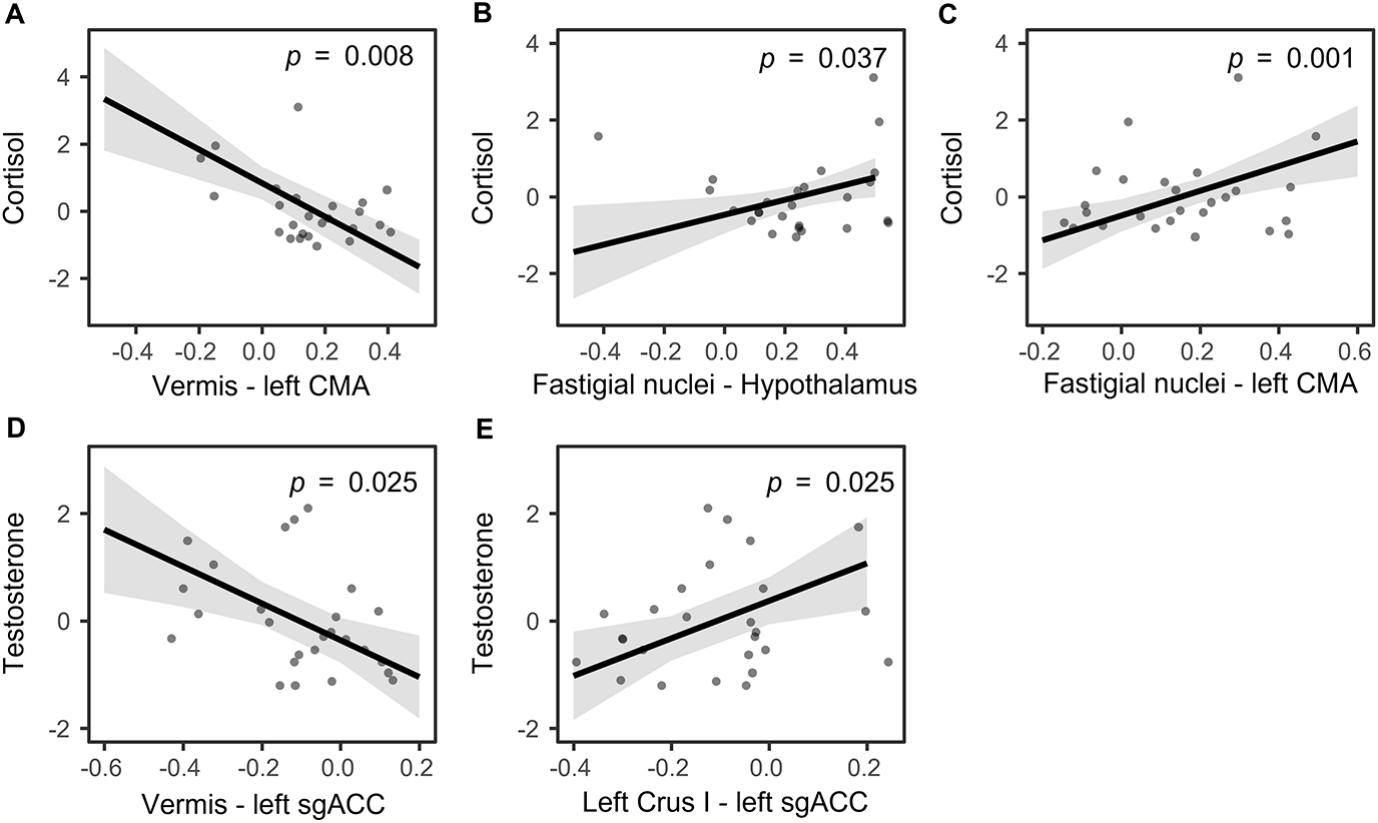
Significant associations between resting-state cerebello–limbic functional connectivity and cortisol (A-C) and testosterone (D-E). Shaded areas illustrate standard errors. Each point indicates one participant.

Significant associations between steroid hormone levels and functional connectivity are shown inFigure 3. Higher cortisol levels were associated with higher functional connectivity between the fastigial nuclei and left CMA (*β *= 3.22,*SE *= 0.93,*t *= 3.45,*p *= 0.008;Fig. 3A) and hypothalamus (*β *= 1.94,*SE *= 0.76,*t *= 2.57,*p *= 0.037;Fig. 3B). A negative association with cortisol levels was found with functional connectivity between the vermis and left CMA (*β *= -4.99,*SE *= 1.08,*t *= -4.64,*p *= 0.001;Fig. 3C). Higher testosterone levels were associated with reduced negative functional connectivity between the functional region in left Crus I and left sgACC (*β *= 3.49,*SE *= 1.23,*t *= 2.84,*p *= 0.025;Fig. 3D) and higher negative functional connectivity between the vermis and left sgACC (*β *=-3.43,*SE *= 1.09,*t *= -3.14,*p *= 0.025;Fig. 3E). For the analysis including T/C ratio, one outlier was removed (Cook’s distance > 0.71). No significant associations for T/C ratio were observed (*p*s > 0.079).

#### 3.2.3 Task-based aggressive behaviour

**Fig. 4.**
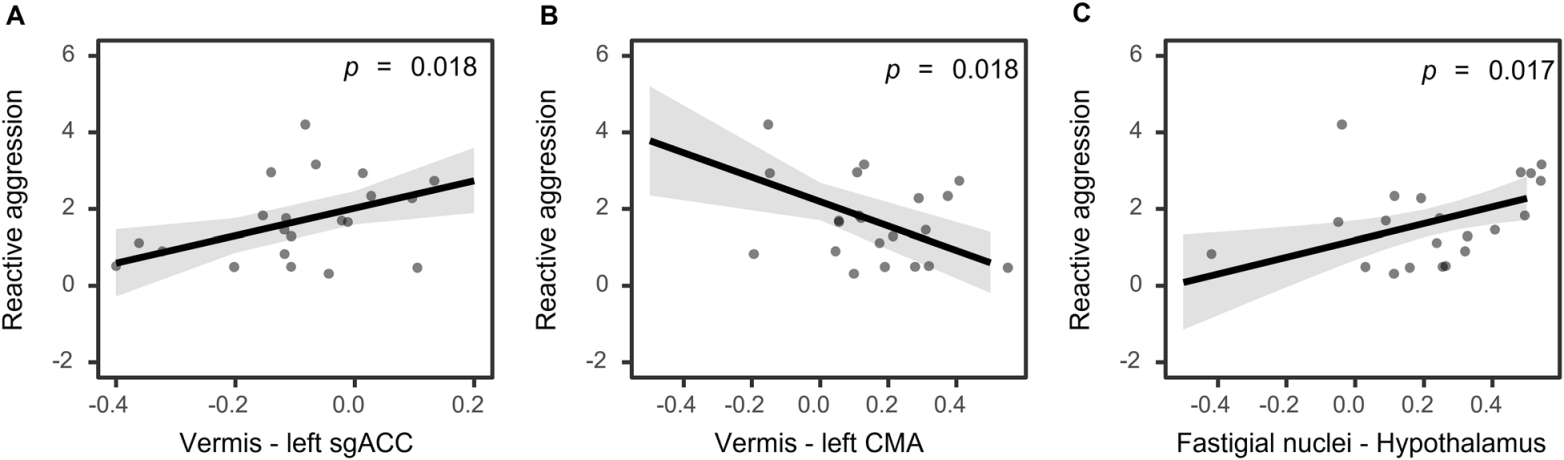
Significant relationships between resting-state cerebello–limbic functional connectivity and task-based aggressive behaviour (A-C). Shaded areas illustrate standard errors of the mean. Each point indicates one participant.

Significant associations between task-based aggressive behaviour and functional connectivity are shown inFigure 4. For the model including reactive aggression (i.e., stealing points following a provocation by the opponent), one participant was removed as an outlier (Cook’s distance > 0.87). More reactive aggression was associated with reduced negative functional connectivity between the vermis and the left sgACC (*β *= 3.58,*SE *= 1.34,*t *= 2.68,*p *= 0.018;Fig. 4A) and increased functional connectivity between the fastigial nuclei and hypothalamus (*β *=2.18,*SE *=0.83,*t *=2.63,*p *=0.018;Fig. 4B). By contrast, a negative association was observed between reactive aggression and functional connectivity between the vermis and left CMA (*β *=-3.17,*SE *=1.07,*t *=-2.97,*p *=0.017;Fig. 4C). No significant results were found for proactive aggression (*p *=0.168).

**Fig. 5.**
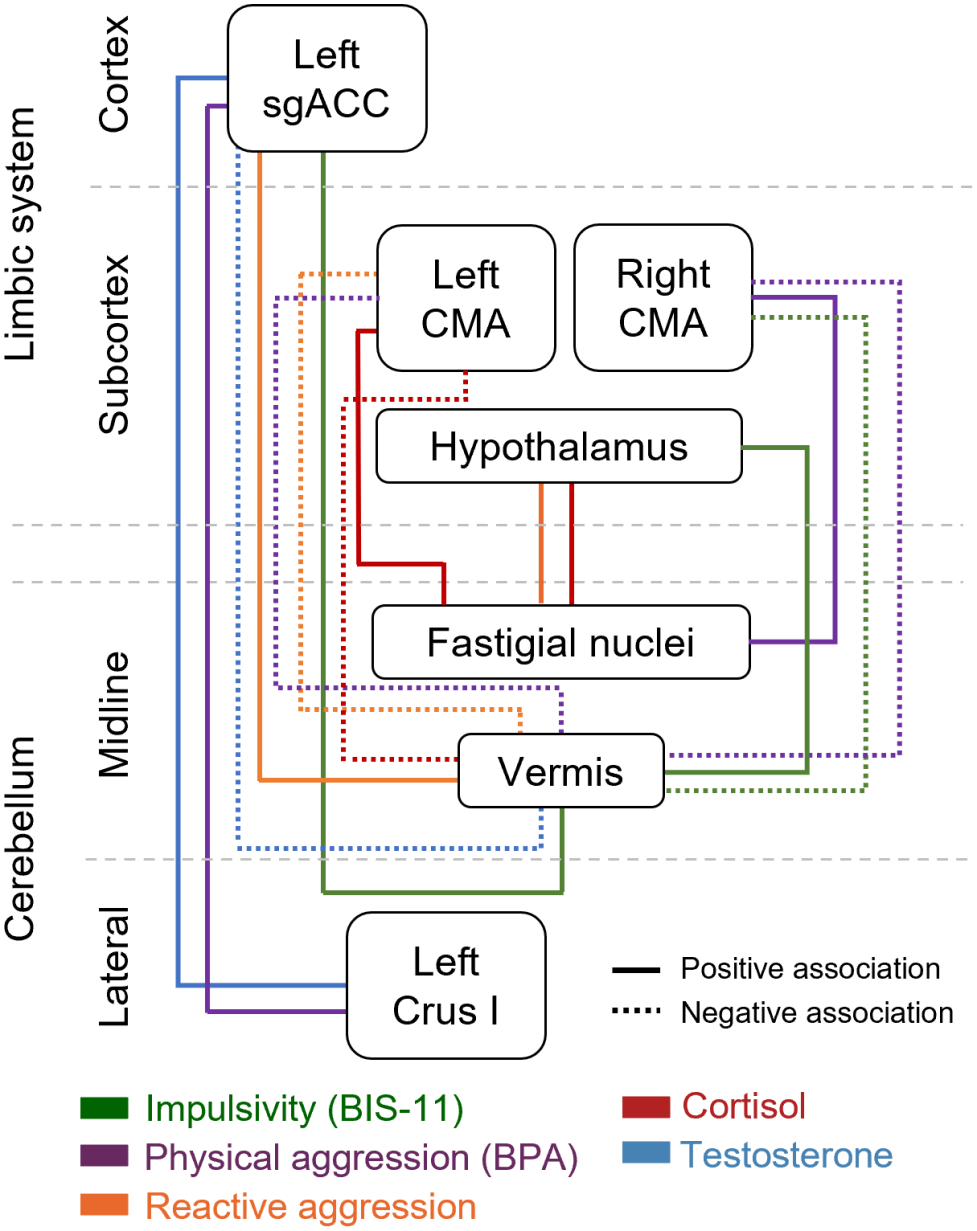
Overview of observed associations between self-reported task-based, and hormone measures and functional connectivity between the cerebellum and the limbic system.

Figure 5depicts a schematic summary of the significant associations between static resting-state cerebello–limbic connectivity, trait physical aggression and impulsivity, steroid hormones, and task-based aggression.

## 4 Discussion

The structural connections between the vermis and limbic circuit via the FN in the mammalian brain (Çavdar et al., 2020,^2022^;Haines et al., 1984) provide a neuro-anatomical blueprint for the observed functional connectivity of the posterior vermis and FN with the bilateral CMA and hypothalamus. Findings of optogenetic stimulation and inhibition of the posterior vermis reducing and increasing aggressive behaviour, respectively (Jackman et al., 2020), concur with reported neural responses in the paraventricular and lateral hypothalamus after electrically stimulating the posterior vermis in dogs (Anand et al., 1959). These outcomes also fit the current association between higher vermis–hypothalamic functional connectivity and higher levels of impulsivity. The vermis has efferent projections to the FN, and intra- and extracellular recording studies in mammals have demonstrated responses in the hypothalamic paraventricular and lateral nuclei after stimulation of the FN in rats (Katafuchi & Koizumi, 1990;Min et al., 1989). The efferent vermis–FN connections extend previous findings of autonomic and aggressive responses to direct electric stimulation of the FN in cats (Reis et al., 1973;Zanchetti & Zoccolini, 1954). The currently observed associations of FN–hypothalamic functional connectivity with cortisol and reactive aggression may hint at a possible link between stress sensitivity and agitated mood states. Indeed, the positive association between basal cortisol levels and FN–hypothalamus functional connectivity concurs with the proposed contribution of the cerebellum to the brain’s stress axis and mood disorders (Schutter, 2016). Patients with major depressive disorder treated with selective serotonergic reuptake inhibitors show a reduction in cerebello–hypothalamic functional connectivity (Yang et al., 2014), which is perhaps indicative of an antidepressant response. Our findings are in agreement with the view that cerebello–hypothalamic connections are part of a neural circuit involved in governing bodily homeostatic processes and associated behaviours (Schutter, 2016;Wen et al., 2004;Zhu et al., 2006).

Earlier work showing responses of the amygdala to intracranial electric stimulation of the vermis in dogs (Anand et al., 1959) exemplifies the existence of signalling routes between the medial parts of the cerebellum and limbic brain regions including connections between the vermis and sgACC. Research using task-based functional connectivity found evidence for higher functional connectivity between the anterior vermis and the left amygdala during the elicitation of disgust (Schienle & Scharmüller, 2013). Furthermore, higher functional connectivity between the vermis and bilateral amygdala during an emotion intervention for traumatic stress symptoms (Monti et al., 2018) is also suggestive for a role in autonomic arousal and learned fear responses (Fastenrath et al., 2022). More specifically, our results show that the vermis is functionally connected to the CMA, but not the BLA. The latter finding contradicts earlier work in animals showing neural responses in the BLA following electric stimulation of the FN and vermis (Snider & Maiti, 1976). By contrast, the functional connection between the vermis and CMA concurs with a recently proposed theoretical framework which postulates that these regions are involved in impulsive and reactive forms of aggressive behaviour (Kruithof et al., 2022;Terburg et al., 2024). These behaviours typically involve more primitive and stereotypical responses following threat or frustration and are argued to be closer tied to intrinsic subcortical network activity. Conversely, the BLA is more intricately connected to the prefrontal cortex and thought to be involved in goal-directed and proactive behaviour (Terburg et al., 2024). These behaviours imply a mentally active state which, next to emotion regulation, requires reasoning and planning. The absence of functional connections involving the BLA suggests that resting-state activity may be less sensitive to capture this type of brain–behaviour relations. This may also explain why we did not observe any relations with task-based proactive aggression. By contrast and in addition to the earlier mentioned hypothalamus, we also observed significant correlations between self-reported impulsivity and vermis–right CMA functional connectivity. These findings extend our previous observation of a positive link between grey matter volume of the posterior vermis and impulsivity in non-clinical volunteers (Wolfs, Klaus, et al., 2023). Furthermore, individual levels of reactive aggression, operationalized as steals in response to provocation during task-based fMRI (Wolfs, van der Zwaag, et al., 2023), were associated with significant resting-state functional connectivity between the FN and hypothalamus, as well as between the vermis and left CMA. As impulsivity is considered a facilitator of reactive aggression, higher resting-state connectivity in the vermis–fastigial–limbic connections may reflect one’s motivational tendency to behaviourally respond in a fast and automatic manner when being provoked. Involvement of the medial cerebellum in autonomic (visceral) activity and the sgACC in monitoring emotional arousal and autonomic states (Schmahmann et al., 2022) is supported by the significant associations of resting-state activity between the vermis and left sgACC. Since higher cortisol and testosterone levels were associated with lower functional connectivity between the vermis and left sgACC, reactive aggression may be linked to a decrease in monitoring emotional arousal, thereby facilitating the brain’s fight mode. Moreover, functional connectivity between the basal ganglia and cerebellum contributes to the processing of angry voices (Thomasson, Ceravolo, et al., 2023). Activation of the vermis (VI–VII) and more lateral hemispheric lobules IV–VI and VIII during vocal emotion processing suggests that the cerebellum together with the basal ganglia also plays a role in vocal decoding and affective responses to vocalized aggression (Ceravolo, Frühholz, et al., 2021).

Physical aggression was correlated with reduced functional connectivity between the vermis and bilateral CMA. Lower resting-state connectivity between the posterolateral cerebellum and amygdala has been found in adult volunteers who report high psychological stress (Kim et al., 2024). While we did not observe any significant associations between Crus I-II and the CMA, the lower functional vermis–CMA connectivity in physical as well as reactive aggression may hint at a possible neural connection between stress and aggressive behaviour. This is supported by findings showing that damage to the vermis or transient functional perturbation of the medial cerebellum with transcranial magnetic stimulation can disrupt emotion regulation (Schmahmann & Sherman, 1998;Schutter & van Honk, 2009). The fact that reactive aggression was associated with reduced functional connectivity between the vermis and left sgACC in conjunction with higher functional connectivity of the FN with the hypothalamus suggests an intrinsic neural bias to more readily engage in aggressive behaviour during provocation. The higher basal levels of testosterone correlating with lower vermis–left sgACC functional connectivity may reflect an instance of cerebello–cortical decoupling underlying reductions in impulse regulation and increased approach motivation. Lower resting-state activity of the left sgACC in this region has, for example, been linked to higher impulsivity in children with attention deficit hyperactivity disorder (Zhan et al., 2017). Finally, the correlation between cortisol and functional cerebellum–amygdala connectivity adds to the proposed role of the medial cerebellum in threat responses through its white matter connections to the amygdala and hypothalamus (Çavdar et al., 2020,^2022^;Dietrichs et al., 1994;Jung et al., 2022). Impulsivity and aggressive behaviour may be thus linked to increased sensitivity of the cerebello–subcortical stress circuit. In line with reported connections between Crus I–II and the prefrontal cortex in primates (Kelly & Strick, 2003;Middleton & Strick, 2001) and humans (Bernard et al., 2016), resting-state functional connectivity between the functional regions in left Crus I and right Crus II and the left sgACC was observed in the current study. The sgACC is implicated in emotional reward value and emotional conflict evaluation (Stevens et al., 2011). Resting-state functional connectivity between bilateral sgACC and the default mode network (DMN) has been linked to higher behavioural activation system scores in adolescents at risk for psychopathology (Iadipaolo et al., 2017), and cerebellar stroke patients with elevated depression levels show reduced functional connectivity in the DMN (Duttagupta et al., 2024). Moreover, functional connectivity of the left Crus I and left sgACC correlated with physical aggression and basal levels of testosterone. Meta-analytic results show that left Crus I is active during threat processing and reward anticipation (Klaus & Schutter, 2021;Kruithof et al., 2023). The functional coupling between Crus I and sgACC correlating with physical aggression and testosterone levels may be part of an internal sensorimotor prediction model involved in maintaining or increasing social status or dominance. Speculatively, the negative connectivity observed in the current study may reflect a decoupling between the posterolateral cerebellar regions related to social and cognitive aspects of aggression and the left sgACC which is linked to mood regulation.

Interestingly, no brain–behaviour associations were found for the connection between right Crus II and the left sgACC. By contrast, the connection between left Crus I and left sgACC was shown to increase (i.e., going from negative to positive functional connectivity) with higher levels of testosterone and self-reported physical aggression, respectively. These findings mirror the negative correlations observed between vermis–sgACC connectivity and testosterone and physical aggression, suggesting a distinct role of left Crus I in this intrinsic aggression circuit. That is, higher testosterone and physical aggression were linked to reduced vermis–sgACC connectivity but increased Crus I–sgACC connectivity. An intrinsic bias towards reactive aggression may thus be supported by the coordinated activity of medial prefrontal and left posterolateral cerebellar regions.

Lastly, the vmPFC is considered an area involved in voluntary goal-directed planning and behaviour (Holton, van Opheusden, et al., 2025). The anticipation of a future steal by the opponent could, for example, be an incentive for the participant to proactively aggress and steal a point in order to demotivate the opponent to do the same. However, contrary to our expectations, no functional connections between the cerebellum and vmPFC were observed. A dysfunctional vmPFC is suggested to play a role in mental illnesses (Hiser & Koenigs, 2018), and abnormalities in cerebello–vmPFC resting-state connectivity have been reported in major depressive disorder (Ma et al., 2013). This could perhaps (in part) explain the difficulty of engaging in goal-directed (proactive) behaviour seen in major depressive disorder and other mental illnesses. The fact that our current sample consisted of a non-clinical population may have limited the sensitivity to detect functional connections between the cerebellum and vmPFC in the context of impulsivity and aggression. Also, the complexity and polysynaptic nature of the cerebello–prefrontal cortical connections may have contributed to the present null finding.

Several study limitations should be considered. First, the functional meaning of anticorrelations remains unclear and is subject to considerable scientific debate. It has been suggested that the inverse correlations reflect competition or modulation effects among regions or networks. The positive correlations between the FN as opposed to the inverse correlations of the vermis with the hypothalamus, CMA, and sgACC, for example, could theoretically be explained by the inhibitory effect of Purkinje cells on the fastigial nucleus. Conversely, the anticorrelation of the vermis to the limbic regions could then be interpreted as an active inhibitory process on the deep cerebellar nuclei (DCN). The DCN consists of the FN, interposed (IN) and dentate nuclei (DN) which relay signals from the cerebellar cortex to the extracerebellar brain regions via excitatory projections. Even though the majority of the efferent vermis connections project onto the FN, connections also run to the IN and DN, which could then explain the anticorrelation as the net outcome of the inhibitory effect of the vermis on the forebrain areas. Second, the relatively small sample size and lower signal-to-noise ratio in subcortical brain areas increase the possibility of false positives (Type I error) and false negatives (Type II error). The brain–behaviour associations found for reactive aggression, for example, were based on only 22 participants as the other eight participants did not steal at all following provocation. Third, functional connectivity analyses involving the hypothalamus were limited to the dorsomedial nucleus and parts of the lateral hypothalamus and paraventricular nucleus (Billot et al., 2020). While the present study used 7T fMRI and universal pulses to optimize cerebellar signals, the hypothalamus remains a challenging region to image due to its proximity to air–tissue boundaries, blood vessels, and CSF. As a result, we did not analyse the ventromedial nucleus although it is considered a critical region in evoking rage and aggression (Hashikawa et al., 2017). Fourth, our cerebellar ROIs are based on task-based activity and while intrinsic network activity is correlated with task-based activity and behavioural indices of aggression, we acknowledge that intrinsic functional connectivity patterns cannot serve as a (latent) substitute for task-based functional connectivity. Fifth, the relationship between functional connections and structural white matter connections should be kept in mind when interpreting resting-state connectivity findings. Some of the current functional connections can be explained by monosynaptic neuroanatomical connections that make up the medial forebrain bundle and the fibres that connect the posterior vermis with the FN and the FN with the hypothalamus (Dietrichs et al., 1994). Still other cerebello–thalamo–cortical connections are polysynaptic. The neuroanatomical plausibility may be relevant to consider when it comes to the sensitivity and specificity of resting-state functional connectivity. Sixth, as a result of our a priori ROI analyses, it is possible that we missed other relevant functional connections. The selection of our ROIs was based on results from a previous structural MRI study (Wolfs, Klaus, et al., 2023), a task-based fMRI study (Wolfs, van der Zwaag, et al., 2023), and a resting-state fMRI study (Wolfs, van Lutterveld, et al., 2023), which all examined associations between the cerebellum and indices of aggression. This approach can be considered a methodological strength as it builds upon previous experimental work. However, potentially relevant cerebellar interactions with regions such as the ventral tegmental area (VTA) are not considered (Fritz, Soravia, et al., 2023). While none of our previous MRI studies indicated VTA involvement in connection to the cerebellum in aggression, this does not preclude the potential relevance of cerebello–VTA interactions or functional cerebellar connectivity with other brain regions. Alternatively, using a larger sample, future studies could perform whole-brain analyses to uncover additional nodes within this network. Seventh, it is not possible to draw inferences on the directionality of the functional connections. Eight, our static approach to functional connectivity is insensitive for dynamic and non-linear spatiotemporal connectivity patterns.

In conclusion, to the best of our knowledge this is the first resting-state fMRI study exploring intrinsic functional connections of the cerebellum with the subcortical aggression circuit, steroid hormones, and indices of aggression in healthy volunteers. Our findings indicate that the well-documented cortico-limbic circuit of human aggression should be extended to include the cerebellum. Future studies are warranted to further map out the precise functional significance of the cerebellar connections.

## Supplementary Material

Supplementary Material

## Data Availability

Data and code are stored in Yoda, a data management system hosted by Utrecht University, and can be accessed upon request athttps://doi.org/10.24416/UU01-B35KD0.
