# Supplementary material for: Functional cerebello–cortico–limbic connectivity in aggression: A resting-state 7T fMRI study in healthy volunteers
Source: Imaging Neurosci (Camb). 2025 Jun 20;3:IMAG.a.42. doi: 10.1162/IMAG.a.42 (PMC12319860; doi:10.1162/IMAG.a.42)
Supplement: Supplementary Material [file imag.a.42_supp.pdf]

Supplementary material

S1. Functional connectivity

Table S1. Overview of all ROI-to-ROI functional connectivity measures between the cerebellum, subcortical and cortical structures.

**ROI**	**Connection**	***F*/*T* Statistic**	$p_{uncorrected}$	p_{FDR}
Cerebellum				
Vermis		$F_{2,26}$ = 158.80	< 0.001	< 0.001
	Vermis – FN	T_{27} = 14.93	< 0.001	< 0.001
	Vermis – Left Crus I	T_{27} = 9.97	< 0.001	< 0.001
	Vermis – Hypothalamus	T_{27} = 8.81	< 0.001	< 0.001
	Vermis – Left CMA	T_{27} = 5.37	< 0.001	< 0.001
	Vermis – Right CMA	T_{27} = 3.91	< 0.001	0.002
	Vermis – Left sgACC	T_{27} = -3.53	0.002	0.004
FN		$F_{2,26}$ = 110.65	< 0.001	< 0.001
	FN – Vermis	T_{27} = 14.93	< 0.001	< 0.001
	FN – Hypothalamus	T_{27} = 6.11	< 0.001	< 0.001
	FN – Right CMA	T_{27} = 5.76	< 0.001	< 0.001
	FN – Left CMA	T_{27} = 4.38	< 0.001	< 0.001
Left Crus I		$F_{2,26}$ = 47.96	< 0.001	< 0.001
	Left Crus I – Vermis	T_{27} = 9.97	< 0.001	< 0.001
	Left Crus I – Left sgACC	T_{27} = -3.50	0.002	0.004
Right Crus II		$F_{2,26}$ = 3.52	0.045	0.045
	Right Crus II – Left sgACC	T_{27} = -2.93	0.007	0.012
Prefrontal cortex				
Left sgACC		$F_{2,26}$ = 88.37	< 0.001	< 0.001
	Left sgACC – Left vmPFC	T_{27} = 10.44	< 0.001	< 0.001
	Left sgACC – Right sgACC	T_{27} = 7.41	< 0.001	< 0.001
	Left sgACC – Right vmPFC	T_{27} = 6.97	< 0.001	< 0.001
	Left sgACC – Vermis	T_{27} = -3.53	0.002	0.003
	Left sgACC – Left BLA	T_{27} = 3.40	0.002	0.008
	Left sgACC – Left Crus I	T_{27} = -3.50	0.002	0.010
	Left sgACC – Right Crus II	T_{27} = -2.93	0.007	0.081
Left vmPFC		$F_{2,26}$ = 71.56	< 0.001	< 0.001
	Left vmPFC – Left sgACC	T_{27} = 10.44	< 0.001	< 0.001
	Left vmPFC – Right vmPFC	T_{27} = 7.40	< 0.001	< 0.001
	Left vmPFC – Right sgACC	T_{27} = 3.94	< 0.001	0.002
Right vmPFC		$F_{2,26}$ = 71.53	< 0.001	< 0.001
	Right vmPFC – Right sgACC	T_{27} = 7.47	< 0.001	< 0.001
	Right vmPFC – Left vmPFC	T_{27} = 7.40	< 0.001	< 0.001
	Right vmPFC – Left sgACC	T_{27} = 6.97	< 0.001	< 0.001
Right sgACC		$F_{2,26}$ = 50.96	< 0.001	< 0.001
	Right sgACC – Left sgACC	T_{27} = 7.41	< 0.001	< 0.001

	Right sgACC – Right vmPFC	$T_{27} = 7.47$	< 0.001	< 0.001
	Right sgACC – Left vmPFC	$T_{27} = 3.94$	< 0.001	0.002
	Right sgACC – Left CMA	$T_{27} = 2.96$	0.006	0.015
Subcortex				
Hypothalamus		$F_{2,26} = 54.04$	< 0.001	< 0.001
	Hypothalamus – Vermis	$T_{27} = 8.81$	< 0.001	< 0.001
	Hypothalamus – FN	$T_{27} = 6.11$	< 0.001	< 0.001
	Hypothalamus – Right CMA	$T_{27} = 4.92$	< 0.001	< 0.001
	Hypothalamus – Right BLA	$T_{27} = 4.85$	< 0.001	< 0.001
	Hypothalamus – Left CMA	$T_{27} = 3.92$	< 0.001	0.002
Right CMA		$F_{2,26} = 53.06$	< 0.001	< 0.001
	Right CMA – Right BLA	$T_{27} = 9.13$	< 0.001	< 0.001
	Right CMA – FN	$T_{27} = 5.76$	< 0.001	< 0.001
	Right CMA – Hypothalamus	$T_{27} = 4.92$	< 0.001	< 0.001
	Right CMA – Vermis	$T_{27} = 3.91$	< 0.001	0.001
Right BLA		$F_{2,26} = 43.31$	< 0.001	< 0.001
	Right BLA – Right CMA	$T_{27} = 9.13$	< 0.001	< 0.001
	Right BLA – Hypothalamus	$T_{27} = 4.85$	< 0.001	< 0.001
	Right BLA – Left BLA	$T_{27} = 5.06$	< 0.001	< 0.001
Left BLA		$F_{2,26} = 40.50$	< 0.001	< 0.001
	Left BLA – Left CMA	$T_{27} = 6.72$	< 0.001	< 0.001
	Left BLA – Right BLA	$T_{27} = 5.06$	< 0.001	< 0.001
	Left BLA – Left sgACC	$T_{27} = 3.40$	0.002	0.004
Left CMA		$F_{2,26} = 27.83$	< 0.001	< 0.001
	Left CMA – Left BLA	$T_{27} = 6.72$	< 0.001	< 0.001
	Left CMA – Vermis	$T_{27} = 5.73$	< 0.001	< 0.001
	Left CMA – FN	$T_{27} = 4.38$	< 0.001	< 0.001
	Left CMA – Hypothalamus	$T_{27} = 3.92$	< 0.001	0.001
	Left CMA – Right sgACC	$T_{27} = 2.96$	0.006	0.019

Abbreviations: BLA = Basolateral Amygdala; CMA = Ventromedial Amygdala; FN = Fastigial Nuclei; sgACC = subgenual Anterior Cingulate Cortex; vmPFC = ventromedial Prefrontal Cortex.

S2. Brain-behaviour-hormone associations

S2.1. Trait impulsivity and aggression

Table S2. Associations between self-reported impulsivity and functional connectivity measures (*N*=28).

Predictors	*β*	*SE*	*CI*	*t*	*p*
Vermis – Left sgACC	25.01	8.87	6.67 – 43.35	2.82	0.024
Vermis – Hypothalamus	18.11	7.46	2.68 – 33.54	2.43	0.039
Vermis – Right CMA	-19.72	9.10	-38.53 – -0.90	-2.17	0.051
FN – Hypothalamus	-12.07	6.81	-26.16 – 2.02	-1.77	0.090
R^2 / R^2 adjusted	0.446 / 0.349				

Table S3. Associations between self-reported physical aggression and functional connectivity measures (*N*=28).

Predictors	*β*	*SE*	*CI*	*t*	*p*
Vermis – Left CMA	-15.30	5.95	-27.64 – -2.95	-2.57	0.026
Vermis – Right CMA	-22.11	6.96	-36.55 – -7.68	-3.18	0.009
FN – Right CMA	27.91	7.12	13.14 – 42.68	3.92	0.002
Left Crus I – Left sgACC	13.25	5.64	1.56 – 24.95	2.35	0.034
Right Crus II – Left sgACC	7.70	4.63	-1.91 – 17.31	1.66	0.111
R^2 / R^2 adjusted	0.498 / 0.384				

S2.2. Steroid hormones

Table S4. Associations between testosterone levels and functional connectivity measures (N=26).

Predictors	*β*	*SE*	*CI*	*T*	*p*
Vermis – Left sgACC	-3.43	1.09	-5.69 – -1.16	-3.14	0.025
Vermis – Left CMA	-2.27	1.19	-4.74 – 0.21	-1.90	0.118
FN – Right CMA	1.78	1.20	-0.72 – 4.27	1.48	0.193
Left Crus I – Left sgACC	3.49	1.23	0.93 – 6.05	2.84	0.025
R^2 / R^2 adjusted	0.395 / 0.279				

Table S5. Associations between cortisol levels and functional connectivity measures (N=26).

Predictors	*β*	*SE*	*CI*	*t*	*p*
Vermis – Left sgACC	-1.18	0.89	-3.04 – 0.68	-1.33	0.240
Vermis – Left CMA	-4.99	1.08	-7.24 – -2.75	-4.64	0.001
FN – Hypothalamus	1.94	0.76	0.37 – 3.52	2.57	0.037
FN – Left CMA	3.22	0.93	1.28 – 5.17	3.45	0.008
Right Crus II – Left sgACC	1.25	0.91	-0.64 – 3.14	1.38	0.240
R^2 / R^2 adjusted	0.554 / 0.442				

Table S6. Associations between T/C ratio and functional connectivity measures (N=25).

Predictors	*β*	*SE*	*CI*	*t*	*p*
FN – Right CMA	-60.15	26.88	-115.76 – -4.55	-2.24	0.070
R^2 / R^2 adjusted	0.179 / 0.143				

S2.3. Task-based aggressive behaviour

Table S7. Associations between reactive aggression (i.e., steals following a provocation in the PSAP) and functional connectivity measures (*N*=21).

Predictors	*β*	*SE*	*CI*	*t*	*p*
Vermis – Left sgACC	3.58	1.34	0.76 – 6.40	2.68	0.018
Vermis – Left CMA	-3.17	1.07	-5.43 – -0.92	-2.97	0.017
FN – Hypothalamus	2.18	0.83	0.43 – 3.94	2.63	0.018
R^2 / R^2 adjusted	0.490 / 0.400				

Table S8. Associations between reactive aggression (i.e., steals following a provocation in the PSAP) and functional connectivity measures (*N*=24).

Predictors	*β*	*SE*	*CI*	*t*	*p*
Vermis – Left CMA	-14.63	8.89	-33.12 – 3.87	-1.64	0.168
Vermis – Right CMA	-13.44	9.41	-33.00 – 6.12	-1.43	0.168
R^2 / R^2 adjusted	0.301 / 0.234				

S2.4. Steroid hormones in males and females

Analyses showed no significant sex differences for cortisol ($t(14.9) = 1.70$, $p = .109$), but a significant sex difference for testosterone was observed ($t(23.9) = 7.54$, $p < .0001$). Breaking the significant effects down by sex as depicted in Figure S1 shows that:

- The negative association between cortisol levels and FC of the vermis and left CMA did not differ between sexes (panel A);
- The positive associations we observed were likely driven by the female participants (panels B, C, E);
- The negative association between testosterone levels and FC of the vermis and left sgACC was stronger for the male participants (panel D).

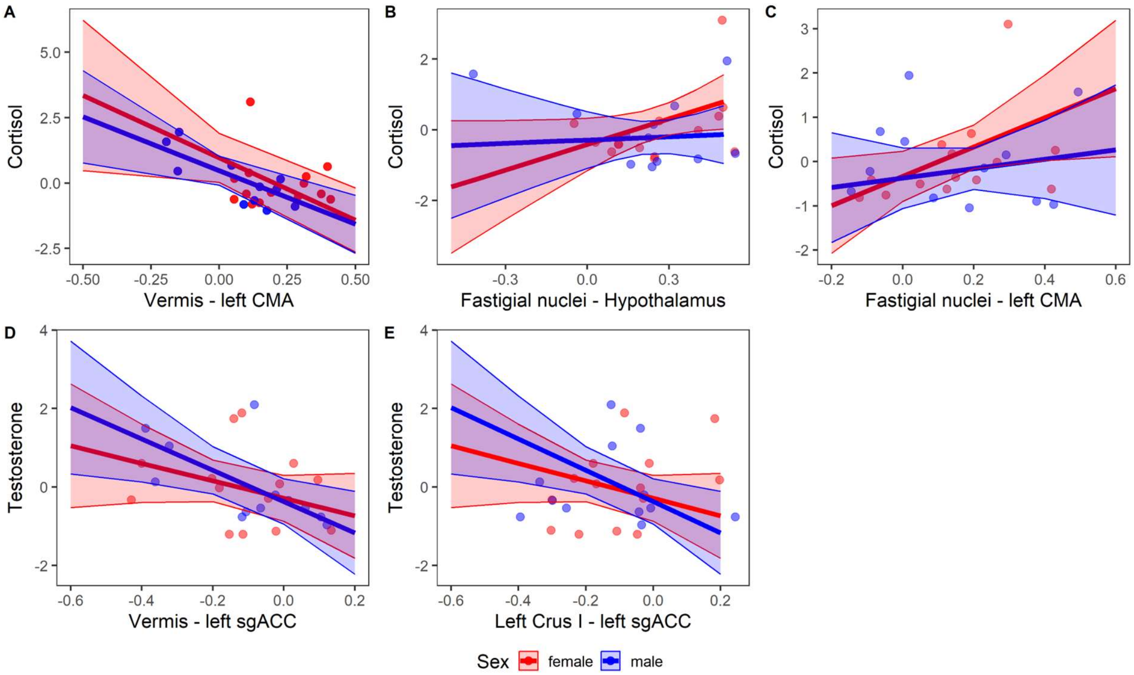

Figure S1. Associations between cerebellar functional connectivity and steroid hormones in females and males.

Note that these are descriptive observations only. Additional models which included the interactions of sex with the respective connectivity measures were also performed. Except for the effect of FC between the vermis and left CMA on cortisol, none of the model terms survived correction for multiple comparisons (though estimates were similar to those from the main models; see Tables S9 and S10). Crucially, however, none of the interaction terms were even close to the critical alpha threshold, which suggests that systematic sex differences on the observed effects are unlikely or could not be detected with our current study design.

Table S9. Associations between individual cortisol levels and functional connectivity measures as a function of sex.

Predictors	*β*	*SE*	*CI*	*t*	p_{FDR}
Sex	-0.18	0.32	-0.86 – 0.51	-0.54	0.790
Vermis - CMA left	-4.44	1.24	-7.03 – -1.84	-3.59	0.017
FN - Hypothalamus	1.36	0.93	-0.59 – 3.31	1.47	0.425
FN - CMA left	2.19	1.13	-0.19 – 4.57	1.93	0.278
Sex * Vermis - CMA left	-0.33	1.24	-2.93 – 2.27	-0.27	0.849

Sex * FN - Hypothalamus	1.05	0.93	-0.90 – 2.99	1.13	0.536
Sex * FN - CMA left	1.12	1.13	-1.26 – 3.50	0.99	0.536
Observations	26				
R^2 / R^2 adjusted	0.559 / 0.387				

Table S10. Associations between individual testosterone levels and functional connectivity measures as a function of sex.

Predictors	*β*	*SE*	*CI*	*t*	p_{FDR}
Sex	0.17	0.22	-0.29 – 0.63	0.76	0.548
Vermis-sgACC left	-3.11	1.13	-5.48 – -0.74	-2.74	0.076
Crus I left - sgACC left	2.51	1.15	0.10 – 4.92	2.17	0.127
Sex * Vermis - sgACC left	0.88	1.13	-1.49 – 3.25	0.78	0.548
Sex * Crus I left - sgACC left	1.18	1.15	-1.23 – 3.59	1.02	0.548
Observations	26				
R^2 / R^2 adjusted	0.364 / 0.205				

S3. Miscellaneous

For an interpretation of the impulsivity levels of our sample, we used results from a norming study by Stanford et al. (2009). An unpaired t-test revealed no significant difference between our sample and the norming sample ($t(28.3) = -0.53$, $p = .601$). Figure S2 illustrates the distribution of our sample along with mean (straight red line) and standard deviations (dashed red lines) and the norming values (blue lines) for reference. Our findings thus fall within an average range of neurotypical, healthy individuals and do not represent pathological levels of impulsivity.

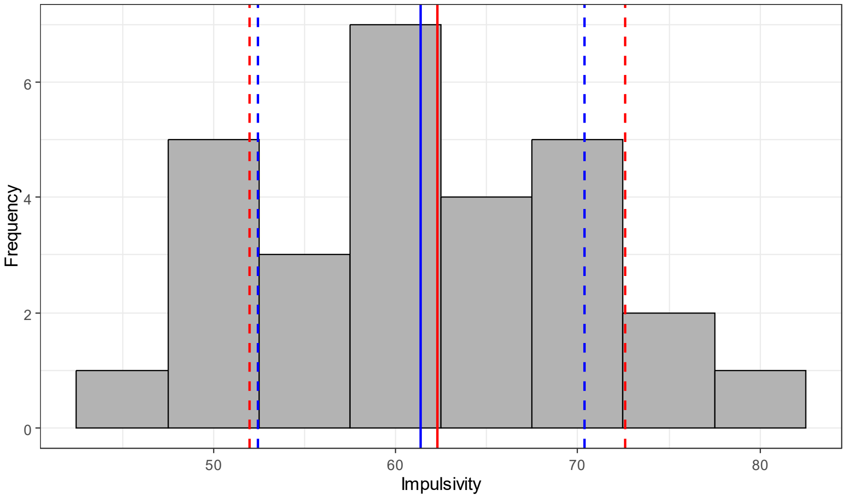

Figure S2. Frequency distribution of self-reported impulsivity as measured by total BIS-11 scores. Red lines indicate values of the current sample (solid = mean; dashed = M ± 1 SD); blue lines indicate values from a reference sample (Stanford et al., 2009; solid lines = mean, dashed lines = M ± 1 SD).

For physical aggression, we used norm values from a study which assessed BPA subscale physical aggression in male Dutch students (Hornsveld et al., 2009). Here, we found a significant difference, with our sample scoring lower on physical aggression than the norming sample ($t(17.21) = -4.63$, $p < .001$; Figure S3). This result remained unchanged when we only included our male participants ($t(46.34) = -4.83$, $p < .001$). Therefore, although no clear cut-off scores exist for the BPA as it is not a diagnostic instrument, our sample seems to fall within a lower range of the student population. Based on the currently used correlational approach, the inclusion of higher aggression and thereby introducing more variation in scores may yield more pronounced effects and even reveal novel associations that we have not been able to detect.

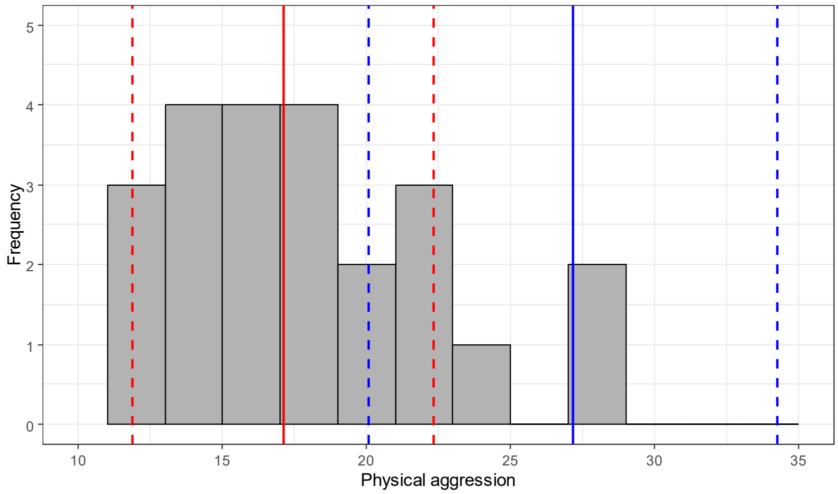

Figure S3. Frequency distribution of self-reported physical aggression as measured by BPA scores (subscale Physical Aggression). Red lines indicate values of the current sample (solid = mean; dashed = *M* ± 1 *SD*); blue lines indicate values from a reference sample (Hornsveld et al., 2009; solid lines = mean, dashed lines = *M* ± 1 *SD*).

Reference values for salivary cortisol measured between 12pm and 4pm were taken from Gagnon, Fréchette, et al. (2018). Because this study reported no standard deviations, but only upper and lower ranges, no statistical comparison between the reference and the current sample can be made. However, looking at Figure S4, the measurements from the current sample fall within the reference range, albeit at the lower end.

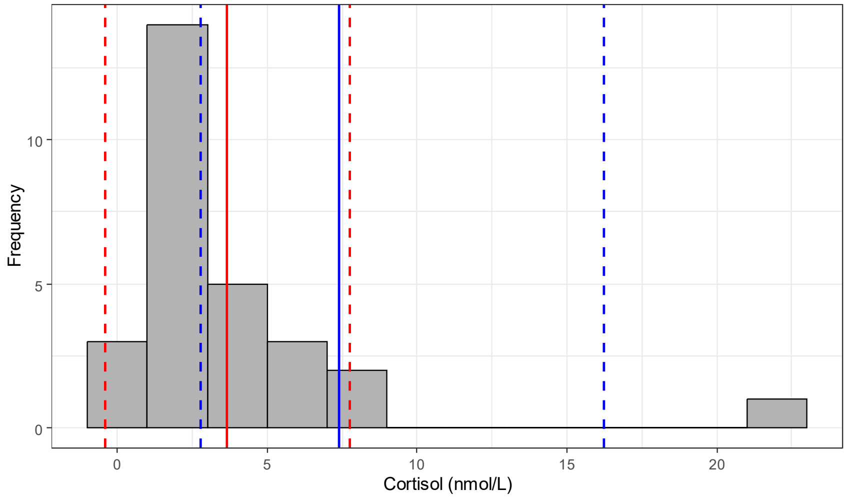

Figure S4. Frequency distribution of individual cortisol levels. Red lines indicate values of the current sample (solid = mean; dashed = *M* ± 1 *SD*); blue lines indicate values from a reference sample (Gagnon et al., 2018; solid line = mean; dashed = range).

Finally, for testosterone, reference values were taken from Keevil, MacDonald, et al. (2013). Testosterone levels of males fall within the ranges of reference (t(71.9) = 1.23, p = .224), while testosterone levels in our female sample were above the range of the reference population (t(13.3) = 6.77, p < .001; Figure S5). We currently have no explanation for this difference, and it would be interesting to investigate whether females who fall within the normal range of testosterone levels show different resting-state patterns.

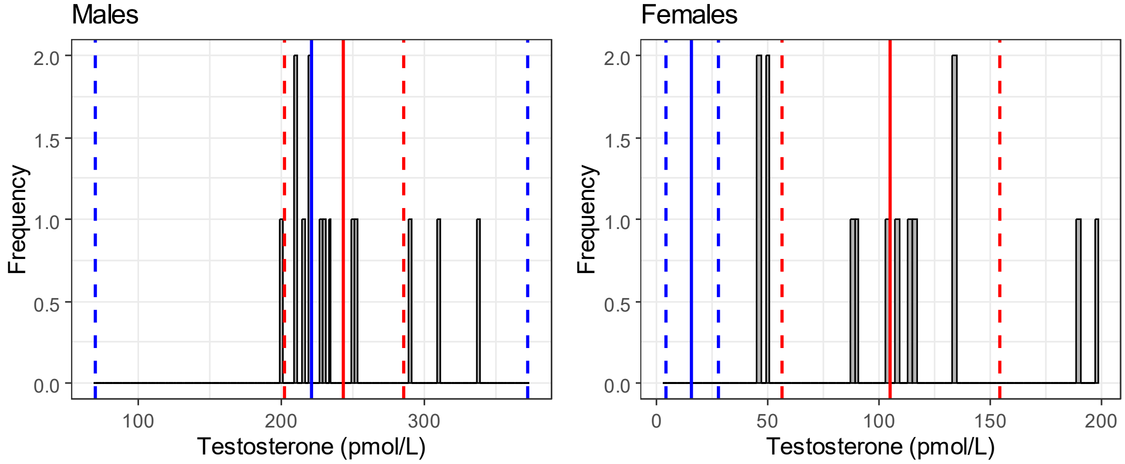

Figure S5. Frequency distribution of individual testosterone levels broken down by sex. Red lines indicate values of the current sample (solid = mean; dashed = *M* ± 1 *SD*); blue lines indicate values from a reference sample (Keevil et al., 2013; solid line = mean; dashed = *M* ± 1 *SD*).

References

Gagnon, N., Fréchette, I., Mallet, P. L., Dubé, J., Houde, G., & Fink, G. D. (2018). Establishment of reference intervals for the salivary cortisol circadian cycle by electrochemiluminescence (ECLIA) in healthy adults. *Clinical Biochemistry*, *54*, 56-60. doi:10.1016/j.clinbiochem.2018.02.012

Hornsveld, R. H. J., Muris, P., Kraaimaat, F. W., & Meesters, C. (2009). Psychometric properties of the aggression questionnaire in Dutch violent forensic psychiatric patients and secondary vocational students. *Assessment*, *16*(2), 181-192. doi:10.1177/1073191108325894

Keevil, B. G., MacDonald, P., Macdowall, W., Lee, D. M., Wu, F. C., & NATSAL Team (2014). Salivary testosterone measurement by liquid chromatography tandem mass

spectrometry in adult males and females. *Annals of Clinical Biochemistry*, *51*(Pt 3), 368-378. doi:10.1177/0004563213506412

Stanford, M. S., Mathias, C. W., Dougherty, D. M., Lake, S. L., Anderson, N. E., & Patton, J. H. (2009). Fifty years of the Barratt Impulsiveness Scale: An update and review. *Personality and Individual Differences*, *47*(5), 385-395. doi:10.1016/j.paid.2009.04.008
